# A proposal for reducing the effect of one of many causes of publication bias

**DOI:** 10.1186/1745-6215-14-41

**Published:** 2013-02-12

**Authors:** Sue M Richards, Julie A Burrett

**Affiliations:** 1 Clinical Trial Service Unit (CTSU), Richard Doll Building, Old Road Campus, Roosevelt Drive, Oxford, OX3 7LF, UK

**Keywords:** Publication bias, Randomized trial, Polycythaemia, Busulphan, Radioactive phosphorous, Venesection

## Abstract

In order to avoid publication bias, all trials should be registered at initiation and their results made easily accessible. However, some trial results are more difficult to publish than others. This report describes one such trial and highlights the need for a way of making results of trials widely available even if not presented in the traditional format. Until such time as it is required by law both to register all trials and enter their final results into the database, a lack of resources will mean that some trial results are never published. The scale of the problem of non-publication is unknown and for valid trial results any form of publication is better than none. Therefore it is essential that a quick and easy way is available to act as a safety net to catch trial results that would otherwise be lost.

## Background

The problem of publication bias in scientific research has been recognized for many years, and was drawn to the attention of the medical community in the 1980s [1]. Since that time many studies have shown that lack, or delay, of publication is related to the statistical significance of the results [2-7]. Awareness of this has been increased by the rise in systematic reviews, and methods to assess the degree of publication bias in these have been developed [8]. These methods provide a rating of the quality of the evidence but do not help in determining a corrected effect estimate.

Some measures have already been put in place to address the problem, such as the requirement that trials must have been registered in a recognized public trials registry at initiation as a condition of consideration for publication in a journal that is a member of the International Committee of Medical Journal Editors [9]. Even for trials which have been registered, publication rates are low [10].

We discuss here one example that illustrates that there may be additional ways of reducing this problem.

## Main text

**Figure 1:**
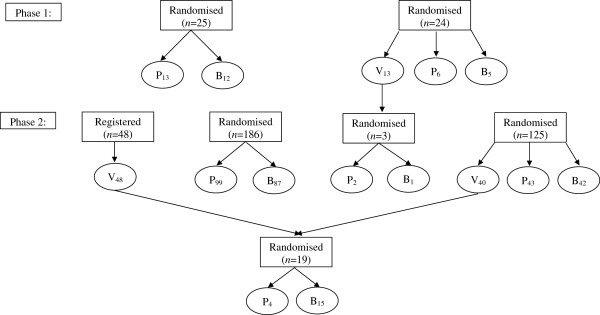
**Randomization structure showing the number of patients allocated to each of the three treatments (Venesection, P32 and Busulphan) and the method of treatment allocation in each phase of the trial.** V_*n1*_, P_*n2*_ and B_*n3*_ indicate that *n*_*1*_ patients were allocated to Venesection, *n*_*2*_ to P32 and *n*_*3*_ to Busulphan.

The Medical Research Council funded a randomized trial in polycythaemia (Figure 1), which recruited between 1974 and 1993, before the days of trial registries. Due to the low mortality rate from this disease, with a median survival of 13 years, follow-up continued until 2003. During this time personnel working on the trial changed, including the departure of the statistician. This trial then came under the remit of the remaining statistician in the Clinical Trial Service Unit responsible for leukemia trials, who reran analyses and wrote a skeleton paper. The introduction, methods and results sections of the paper were drafted, but the discussion section was incomplete and the clinical lead then retired. The computer system on which the analysis programs ran has now been superseded. Although programs and data have been archived it would take a considerable amount of work to do any further analyses.

The paper remains without an abstract, discussion section or references and there are no resources available for any further work to be done. The trial was completed and we strongly believe that the results should be made publicly available. However, we have not found any journal that would accept the paper in this format (see Additional file 1: Medical Research Council randomized Polycythaemia trial results: long term outcome after busulphan, radioactive phosphorous or venesection).

## Discussion

Much recent discussion has focused on the issue of competing, particularly financial, interests, and the role of the pharmaceutical industry. This has led to a new US law requiring both the registration of trials and the entry of final results into a database [11], and the suggestion that legislation should be expanded internationally [12]. However, there are other reasons behind non-publication, including a lack of resources, as in the example presented here.

## Conclusions

The scale of the non-publication of trials is unknown, but providing a medium for reporting unpublished trials, together with any results that are available from them, would provide further information on this subject.

## Competing interests

The authors declare that they have no competing interests.

## Authors’ contributions

SR wrote the first draft of the commentary, JAB revised it, and both authors read and approved the final manuscript.

## Supplementary Material

Additional file 1 Medical Research Council randomized Polycythaemia trial results: long term outcome after busulphan, radioactive phosphorous or venesection. Click here for file
