# Supplementary material for: A proposal for reducing the effect of one of many causes of publication bias
Source: Trials. 2013 Feb 12;14:41. doi: 10.1186/1745-6215-14-41 (PMC3598957; doi:10.1186/1745-6215-14-41)
Supplement: Additional file 1 — Medical Research Council randomized Polycythaemia trial results: long term outcome after busulphan, radioactive phosphorous or venesection. [file 1745-6215-14-41-S1.doc]

**Medical Research Council randomized Polycythaemia trial results: long term outcome after busulphan, radioactive phosphorous or venesection.**

Authors

Sue Richards (retired) ^1^

Jill Durrant (retired) ^1^ Email: jilldurrant@doctors.org.uk

Richard Peto^1^ Email: [secretary@ctsu.ox.ac.uk](mailto:secretary@ctsu.ox.ac.uk)

^1^Clinical Trial Service Unit (CTSU), Richard Doll Building, Old Road Campus, Roosevelt Drive, Oxford OX3 7LF

**ABSTRACT**

**Background**

Polycythaemia is a myeloproliferative disorder in which too many red blood cells are produced in the bone marrow. In the 1970s several treatments were available but there were no good studies evaluating them.

**Methods**

In 1974 the UK Medical Research Council initiated a randomised trial comparing venesection, oral busulphan and intravenous radioactive phosphorous (P32). Initially patients were all randomised either between P32 and busulphan, or between all three treatments. After a major amendment in 1976, patients could be registered and treated with venesection. These patients, as well as those initially randomised to venesection, could be randomised between P32 and busulphan when disease control was lost. Recruitment closed in 1993 and follow-up continued to 2003.

**Results**

408 patients were eligible for analysis. Overall median survival was 12 years. There was no significant difference in survival between those treated actively with P32 or busulphan compared with those venesected; odds ratio (OR) = 1.03 (95% confidence interval (CI) = 0.69-1.52). Busulphan gave superior survival to P32; OR=0.68 (95% CI=0.52-0.89; *p*=0.005). There was no evidence of an excess in bone marrow failure and neoplasms with busulphan.

**Conclusions**

These results suggest that although 32P and Busulphan control the disease process they do not induce leukaemic transformation or bone marrow failure. Busulphan used intermittently at a relatively low dose (4mgs. daily) together with a follow-up interval of 6 months or more is effective and cheap.

**KEYWORDS**

randomised trial, polycythaemia, busulphan, radioactive phosphorous, venesection

**MAIN TEXT**

**Background**

Polycythaemia vera is a myeloproliferative disorder in which too many red blood cells are produced by the bone marrow, without any obvious cause. Some people may have a faulty gene, called the JAK2 gene, that might have caused their condition. Having too many red blood cells results in a raised level of haemoglobin. Too many red blood cells can make the blood much thicker than normal. Some people with polycythaemia also have increased numbers of white cells and platelets in their blood. Like other myeloproliferative diseases, polycythaemia is a rare condition. It mainly occurs in adults aged over 40, and is rare before this age. The causes are unknown.

In the 1970’s several treatments for polycythaemia were available, but there were no good studies evaluating them. In 1974 the Medical Research Council (MRC) initiated a randomised trial for patients with a diagnosis of primary proliferative polycythaemia comparing venesection (V), oral busulphan (Bu) and intravenous radioactive phosphorous (P32). Randomisation could be between Bu and P32 alone (2-way) or between V, Bu and P32 (3-way), according to patient and clinician preference. Recruitment was very slow and it was becoming apparent that many patients were being managed with venesection together with oral aspirin to counteract thrombotic events. At this point one of the original co-ordinators Dr. Leon Szur sadly died and the trial co-ordination was passed to Dr Jill Durrant of this paper. Also a decision was made by the Medical Research Council to relocate the administrative centre for their trials from The Royal Marsden Hospital to Oxford. Professor Sir Richard Peto was involved in re-vamping the trial-known as the “new trial” which opened in 1976. This allowed additional patients to be registered if they were to be treated with venesection, with the possibility of entering the 2-way randomisation later at the time they required more active treatment. Patients allocated V in the 3-way randomisation could also be randomised later in the 2-way.

**Methods**

Eligibility to the trial was by appropriate clinical and laboratory investigation. Patients were excluded if they had been previously treated with any cytotoxic agent or had a life-threatening inter-current disease. Patients who had been venesected or had received anti-thrombotic drugs were included. Initial treatment was by venesection to control the haematocrit and oral aspirin or another anti-thrombotic agent of the physician’s choice, to prevent thrombosis.

The trial recruited between February 1974 and April 1993, with a major amendment in 1976. In the first part of the trial patients could be randomised between P32 and Bu (2-way) or V, P32 and Bu (3-way). After the amendment, patients could be registered without randomisation (registration), managed with venesection until disease control was lost i.e. there was a rapidly rising blood-cell count or hepato-splenomegaly or severe iron deficiency. At this stage the patient was re-investigated and could then be randomised between the 2 “active” treatments (2-way). Thus, for each patient, the decision was initially made about which treatments might be appropriate and they were entered into the registration, 2-way or 3-way group, while those entered as registration, or allocated venesection in the 3-way, could later move to the 2-way group. The flowchart in Figure 1 shows the number of patients allocated to each of the three treatments and the method of treatment allocation during each phase of the trial. In the initial phase only patients with a platelet count exceeding 500x10^9^/l could be randomised between active treatment (2-way), but this was deemed to be unnecessary when the trial was re-designed.

**Figure 1. Randomisation structure showing the number of patients allocated to each of the three treatments (venesection, P32 and busulphan) and the method of treatment allocation in each phase of the trial.**

V*_n1_*, P*_n2_* and B*_n3_* indicate that *n_1_* patients were allocated to venesection, *n_2_* to P32 and *n_3_* to busulphan.

Oral Busulphan was given in a daily dose of 4-6 mgs for 28 consecutive days. During the initial treatment phase therapy could be continued for longer with extreme caution to avoid marrow hypoplasia. 32P was given as a dose of 15cgy intra-venously at an interval of 6-12 weeks up to a maximum dose of 45 cgy per year. Adjuvant venesection and anti-thrombotic treatment was allowed in both “active” arms.

Randomisation was initially done using paper randomisation lists in a central office, but was transferred on to computer in 1977. We asked no criteria for entry except that a diagnosis of Primary Proliferative Polycythaemia had been made according to standard criteria.We reduced form-filling to a minimum and only followed-up patients annually. Death certificates were sent to us automatically from a central registry for validation.

For the final analysis the results of the two phases of the trials have been analysed together. Publication was delayed until at least 50% of patients had died –the median survival being 12 years from trial entry. Follow-up is to April 2003.

**Results**

A total of 414 patients were entered, of whom 6 were excluded because randomisation information was lost or unclear after transfer of the trial administration to Oxford. 408 patients were entered into the final analysis.

49 patients were entered into the first phase of the trial and 359 after the amendment. Table 1 and Figure 1 show the numbers of patients allocated each treatment, by randomisation type and phase of trial. Of the patients initially treated with venesection, 3 from the first phase, and 19 from the second phase, were randomised later for active treatment, 6 to P32 and 16 to Bu.

Venesection was used as adjuvant treatment in both P32 and Bu arms together with “anti-platelet” therapy the most widely used being 75-150 mgms. of aspirin daily or di-pyrimadole.

Table 2 shows the distribution of age and gender by treatment.

Overall the median survival is 13 years, 11 years and 9 months for those treated actively with Busulphan or P32 and 14 years for those venesected. In the “two-way” randomisation those treated with P32 have a median survival of 10 years and 6 months and with Busulphan 13 years. There is no statistically significant difference in survival between those treated actively with P32 and Busulphan and those venesected; odds ratio (OR) = 1.03 (95% confidence interval (CI) = 0.69-1.52). There is a statistically superior survival for those treated with Busulphan compared with those treated with P32; OR=0.68 (95% CI = 0.52-0.89); *p*=0.005.

The causes of death have been roughly divided into 7 categories (Table 3). Thrombotic deaths include pulmonary embolus, stroke and coronary heart-disease. Deaths due to myelofibrosis, the myelodysplasias and acute leukaemia were grouped together in analyses as bone-marrow failure deaths. We looked to see if an excess of patients have died from infection due to myelosupression in the groups treated actively. We have grouped together all those patients we believe have died from unrelated causes.

There was no significant excess of bone marrow failure and neoplasms 54/146 (37%) and 68/161 (42%) respectively in those treated with Bu as compared to those treated with P32. 14/48 (29%) occured in those who initally elected venesection, and 17/53 (32%) among those randomised to venesection compared to 33/96 (34%) allocated Bu or P32 in the 3-way comparison.

There were 15/146 (10%) infective deaths in the Bu group compared with 12/161 (7%) in the P32 group, 3/48 (6%) in the venesected elected group, and 9/53(17%) in those randomised to venesection compared with 9/96 (9%) in the Bu/P32 arms.

No patients died from haemorrhage which could be directly related to treatment.

No statistically significant differences in outcome were found for V versus active treatment, for survival censoring at unrelated causes (OR=0.98; 95% CI = 0.64-1.45), survival also censoring at infective, bone marrow failure and cancer causes (OR=1.04; 95% CI = 0.63-1.72), or survival censored also at thrombotic death (OR=1.20; 95% CI = 0.58-2.45).

Survival analyses comparing P32 versus Bu for these endpoints were all in favour of P32, but some were not statistically significant. Censored at unrelated causes: OR=0.76 (95% CI = 0.57-1.01); censored also at infective, bone marrow failure and cancer causes: OR=0.78 (95% CI = 0.56-1.01); censored also at thrombotic death: OR = 0.57 (95% CI = 0.36-0.89).

**Competing interests**

All authors declare no competing interests.

**Authors’ contributions**

RP designed the study and planned the analyses. JD provided clinical guidance and helped write the paper. SR performed the final analyses and wrote the first draft.

**Acknowledgements**

We would like to acknowledge the patients and doctors involved in this trial, and all those who worked on the administration and analyses over the years. Funding was from the Medical Research Council.

Table 1. Numbers of patients in each treatment allocation by initial randomisation type and phase of trial.

| Phase | Randomisation type | Treatment allocation | | |
| --- | --- | --- | --- | --- |
|  |  | V | Bu | P32 |
| Initial | 2-way | - | 12 | 13 |
|  | 3-way | 13 | 5 | 6 |
| Revised | Registration | 48 | - | - |
|  | 2-way | - | 87 | 99 |
|  | 3-way | 40 | 42 | 43 |
| Overall | Registration | 48 | - | - |
|  | 2-way | - | 99 | 112 |
|  | 3-way | 53 | 47 | 49 |

16 Bu and 6 P32 initially allocated to V are included only according to their initial treatment

Table 2. Distribution of age and gender by treatment allocation.

|  | 3-way | | 2-way and 3-way | |
| --- | --- | --- | --- | --- |
|  | V | Bu + P32 | Bu | P32 |
| Male | 29 | 62 | 79 | 98 |
| Female | 24 | 34 | 83 | 69 |
| Age<50 | 6 | 17 | 28 | 30 |
| 50-59 | 16 | 18 | 35 | 36 |
| 60-69 | 20 | 39 | 60 | 60 |
| ≥70 | 11 | 20 | 37 | 40 |

Table 3. Causes of death by treatment allocation.

| Cause of death | Registration | 2 or 3-way | | 3-way | | Total^*^ |
| --- | --- | --- | --- | --- | --- | --- |
|  | V | Bu | P32 | V | Bu or P32 |  |
| Thrombotic | 9 | 34 | 27 | 14 | 23 | 83 |
| Infective | 4 | 17 | 12 | 10 | 9 | 41 |
| Myelofibrosis | 7 | 21 | 21 | 5 | 12 | 50 |
| AML | 1 | 17 | 25 | 7 | 7 | 43 |
| Myelodysplasia | 0 | 4 | 0 | 1 | 2 | 5 |
| Other cancer | 6 | 17 | 25 | 4 | 13 | 51 |
| Unrelated cause | 6 | 8 | 22 | 3 | 10 | 39 |
| Unknown | 1 | 3 | 6 | 0 | 2 | 10 |
| Total | 34 | 121 | 138 | 44 | 78 | 322 |

* Some deaths (for patients initially allocated V and later randomised to Bu or P32, and for patients randomised to Bu or P32 in the 3-way randomisation) are included in more than one treatment column but only once in the total.

**Figure 1. Randomisation structure showing the number of patients allocated to each of the three treatments (venesection, P32 and busulphan) and the method of treatment allocation in each phase of the trial.**

Randomised (*n*=25)

Randomised (*n*=24)

Phase 1:

Phase 2:

Randomised (*n*=3)

Registered (*n*=48)

Randomised (*n*=186)

Randomised (*n*=125)

Randomised (*n*=19)

V*_n1_*, P*_n2_* and B*_n3_* indicate that *n_1_* patients were allocated to venesection, *n_2_* to P32 and *n_3_* to busulphan.
